# Biomarkers detected in cord blood predict vaccine responses in young infants

**DOI:** 10.3389/fimmu.2023.1152538

**Published:** 2023-05-12

**Authors:** Carolyn H. Baloh, Guglielmo M. Venturi, Bernard M. Fischer, Liane S. Sadder, Julie J. Kim-Chang, Cliburn Chan, Kristina De Paris, Li Yin, Grace M. Aldrovandi, Maureen M. Goodenow, John W. Sleasman

**Affiliations:** ^1^ Division of Allergy and Immunology, Department of Pediatrics, Duke University School of Medicine, Durham, NC, United States; ^2^ Department of Biostatistics and Bioinformatics, Duke University School of Medicine, Durham, NC, United States; ^3^ Institute of Global Health and Infectious Diseases, School of Medicine, University of North Carolina at Chapel Hill, Chapel Hill, NC, United States; ^4^ Department of Microbiology and Immunology, School of Medicine, University of North Carolina at Chapel Hill, Chapel Hill, NC, United States; ^5^ Molecular HIV Host Interactions Section, National Institute of Allergy and Infectious Diseases, Bethesda, MD, United States; ^6^ Division of Infectious Diseases, Department of Pediatrics, University of California, Los Angeles, CA, United States

**Keywords:** human infants, immune development, A proliferation-inducing ligand (APRIL), macrophage, tetanus vaccine, B cell, lymphoid germinal centers

## Abstract

**Introduction:**

Factors influencing vaccine immune priming in the first year of life involve both innate and adaptive immunity but there are gaps in understanding how these factors sustain vaccine antibody levels in healthy infants. The hypothesis was that bioprofiles associated with B cell survival best predict sustained vaccine IgG levels at one year.

**Methods:**

Longitudinal study of plasma bioprofiles in 82 term, healthy infants, who received standard recommended immunizations in the United States, with changes in 15 plasma biomarker concentrations and B cell subsets associated with germinal center development monitored at birth, soon after completion of the initial vaccine series at 6 months, and prior to the 12-month vaccinations. Post vaccination antibody IgG levels to *Bordetella pertussis*, tetanus toxoid, and conjugated *Haemophilus influenzae* type B (*HiB*) were outcome measures.

**Results:**

Using a least absolute shrinkage and selection operator (lasso) regression model, cord blood (CB) plasma IL-2, IL-17A, IL-31, and soluble CD14 (sCD14) were positively associated with pertussis IgG levels at 12 months, while CB plasma concentrations of APRIL and IL-33 were negatively associated. In contrast, CB concentrations of sCD14 and APRIL were positively associated with sustained tetanus IgG levels. A separate cross-sectional analysis of 18 mother/newborn pairs indicated that CB biomarkers were not due to transplacental transfer, but rather due to immune activation at the fetal/maternal interface. Elevated percentages of cord blood switched memory B cells were positively associated with 12-month *HiB* IgG levels. BAFF concentrations at 6 and 12 months were positively associated with *pertussis* and *HiB* IgG levels respectively.

**Discussion:**

Sustained B cell immunity is highly influenced by early life immune dynamics beginning prior to birth. The findings provide important insights into how germinal center development shapes vaccine responses in healthy infants and provide a foundation for studies of conditions that impair infant immune development.

## Introduction

During the first 6 months of life, healthy newborns in the United States receive 16 vaccinations that include challenge to over 20 different immunizing antigens (1). However, infancy is a time of transient B-cell hypo-responsiveness, and establishing immunologic memory requires multiple booster vaccines to maintain protection during early life (2). Factors that influence the breadth and character of infant immune priming include interactions between innate and adaptive immunity within the lymphoid germinal centers, which transition immune polarization away from fetal and maternal tolerance toward the postnatal environment formed by pathogen exposure and the microbiome (3). Humoral immunity in infants consists predominantly of naïve, IgM^+^ B cells that have not undergone class switch or affinity maturation (4). Germinal centers are underdeveloped due to low levels of T follicular helper cells, decreased expression of Th1 cytokines, and functional immaturity of follicular dendritic cells (5). Beyond germinal centers and T-cell and B-cell development, early vaccine antibody responses are influenced by passively acquired maternal IgG and breastfeeding, mode of delivery, and antibiotic use (6–9).

Adding to this complexity, individual vaccine antigens and vaccine adjuvant formulations vary in their immunogenicity; thus, assessing vaccine responses in infants requires consideration of multiple variables to determine the role that individual factors play in maintaining antibody-mediated protection. Measurable biomarkers of immune development include multiple cell types, along with their associated cytokines and cellular receptors. Germinal center development and B-cell class switch are associated with IL-21, CD40/CD40 ligand, BAFF, and APRIL (10). The role of macrophages can be assessed by measuring soluble CD14 (sCD14), the end result of signaling through TLR4 known to provide additional boost for antibody production as demonstrated by the use of TLR4 agonists as adjuvants to induce germinal center B-cell antibody production (11). Biomarkers of T-cell differentiation include IL-2, IL-17, IL-22, and interferon γ (IFN-γ). Taken together, these biomarkers can be measured in plasma and enable a means to establish bioprofiles of early immune development.

Few studies have examined the multiple factors influencing vaccine responses in healthy infants (2, 3). Consequently, normal ranges for many immune cytokines have not been established in healthy infants over the first year of life to enable comparisons to infants with altered immunity. In our study, the complex network cells and cytokines shaping vaccine responses over time were examined in a cohort of healthy infants to define the relationships between immune biomarkers and sustained vaccine responses at 1 year of age. Changes in plasma concentrations of biomarkers associated with humoral immune development were examined based on the hypothesis that factors associated with B-cell survival and germinal center formation best predict sustained vaccine antibody levels during the first year of life. Bioprofiles were measured at birth; prior to vaccination; soon after the initial vaccine battery at 2, 4, and 6 months; and then 6 months later to determine their relationships to vaccine IgG levels. The study population included a longitudinal assessment of infants who were breast, formula, or mixed fed during the first year of life. These infants were subsequently examined for antibody responses to three T-cell-dependent standard vaccines [*Bordetella pertussis*, tetanus toxoid, and conjugated *Haemophilus influenzae type B* (*HiB*)] with the goal of identifying biomarkers that contribute to sustained antibody levels in healthy infants (12, 13).

## Methods

### Study design

Pregnant or immediately post-partum women were recruited through the obstetrics clinics and the labor and delivery clinics of the University of South Florida, All Children’s Hospital, Tampa, FL; University of North Carolina at Chapel Hill Hospitals, Chapel Hill, NC; and Duke University Medical Center and Health System, Durham, NC. All mothers provided informed consent approved by the Institutional Review Board at each location (ClinicalTrials.gov Identifier NCT00683579). For this longitudinal study, 82 newborns were eligible for enrollment, and only infant samples were obtained. Mothers could elect their preferred feeding methods. Maternal immunization history was not available for all mothers. Exclusion criteria included infants delivered by caesarian section (C-section) or <37 weeks’ gestation; infants born with congenital conditions, such as inborn errors of immunity or metabolic disorders; and infants with chronic medical conditions, particularly those that could influence infant immunity such as maternal HIV, immunodeficiency, use of immunosuppressive medications, malignancy, or autoimmunity. For the purpose of evaluating transplacental transfer of plasma factors, an additional cross-sectional cohort of 18 de-identified, archived paired mother/infant plasma samples were obtained from the Carolina Cord Blood Bank. Maternal samples were collected within 2 days of delivery, and corresponding infants’ cord blood (CB) plasma samples were obtained at birth.

**Figure 1:**
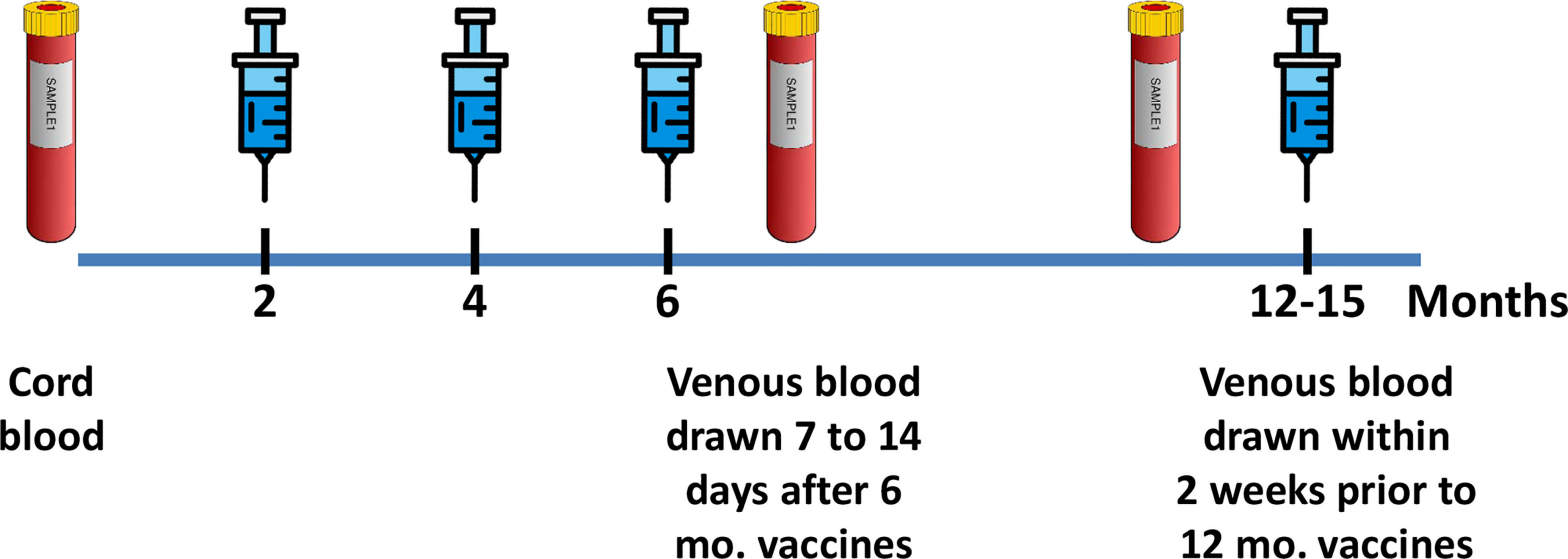
Study design. Longitudinal blood samples, symbolized by blood tubes, were obtained from newborn cord blood, at 7 to 14 days after the 6-month immunization series and at 12 months prior to the immunizations. Blue syringes symbolize the vaccines administered at 2, 4, 6, and 12 months [*Diphtheria*, *tetanus*, acellular *Pertussis*, *Haemophilus influenza*e type b (*HiB*), *Hepatitis* B, *Rotavirus*, and *pneumococcal* conjugate], as recommended by the United States AAP/ACIP guidelines. The 6-month blood sample represents the immediate post-vaccine immune response, and the 12-month sample, drawn prior to the 12-month vaccinations, represents sustained “memory”, IgG antibody concentrations to *Bordetella pertussis* toxin, *HiB, and* tetanus toxoid at 12 months.

Longitudinally assessed infants received standard immunizations on the schedule recommended by the American Academy of Pediatrics (AAP) for all infants in the United States (1). Immunizations included three doses of the *pertussis*, tetanus diphtheria [Pediatrix (DTaP/HepB/IPV): 54.2%, Pentacel (DTaP/IPV/*HiB*): 45.8%, or Daptacel (DTaP): 1.4%], and *HiB* [Pentacel (DTaP/IPV/*HiB*): 45.8%; Pedax (*HiB*): 37.8%, or ActHib (*HiB*): 10.8%] at 2, 4, and 6 months of age. No further immunizations were administered until 12 months of age. When possible, each newborn had a CB sample (pre-immunization) collected. Additional infant peripheral blood samples of 7 to a maximum of 20 ml of whole blood were collected approximately 7–14 days after the 6-month immunization as early vaccine response and within 2 weeks prior to the 12-month vaccinations as the 6-month “memory” response (**Figure 1**). From the whole blood, double-spun plasma aliquots were collected and cryopreserved for batch measurements. Peripheral blood mononuclear cells (PBMCs) were collected by gradient centrifugation with Lymphoprep™ (Stem Cell Technologies, Cambridge, MA). Infants’ missing data from one of the time points were still included in the analysis. Mothers recorded their feeding methods, illnesses, medications, vaccinations, and dates using a personal diary until their infants were 12 months of age. Study data were collected and managed using Research Electronic Data Capture (REDCap) secure electronic data capture tools hosted at Duke University (14, 15). Data recorded included infant sex, race, ethnicity, and maternal Group B Streptococcus (GBS) screening status. Feeding outcomes were classified into three groups: exclusive breastfeeding for 6 months (breast fed), exclusive formula feeding (formula fed), or mixed feeding (mixed fed).

### ELISAs for vaccine responses

The IgG levels to *Bordetella pertussis*, tetanus toxoid, and *Haemophilus influenzae type B (HiB)* were measured according to the respective manufacturer’s instructions: *Bordetella pertussis* (Abcam, Cambridge, MA) (16), tetanus (Binding Site, San Diego, CA), and *Haemophilus influenzae* (Binding Site, San Diego, CA) (13, 17). *Bordetella pertussis* ELISA used *Bordetella pertussis* toxin-coated 96-well plates for detection of IgG (U/ml). Tetanus ELISA used tetanus toxoid antigen-coated 96-well plates for detection of IgG (IU/ml). *Haemophilus influenzae* ELISA used 96-well plates coated with *HiB* capsular polysaccharide antigen conjugated to human serum album for the detection of IgG (mg/ml). ELISA results were analyzed using Tecan’s Magellan™ Software.

### Enumeration of leukocyte populations using multi-color flow cytometry

A complete blood count (CBC) with differential was performed on collected whole blood samples (Beckman Coulter A^c^ Tdiff2, Beckman Coulter, Inc.). Percentages of T cells, B cells, and natural killer cells were measured in 50 µl of whole blood by multi-color flow cytometry using the six-color TBNK reagent (data not shown) (BD, Franklin Lakes, NJ). B-cell subsets were measured in freshly isolated PBMCs by multi-color flow cytometry using the FACS Canto cytometer (BD, Franklin Lakes, NJ). Fluorochrome-conjugated antibodies used for B-cell subset analysis by flow cytometry included CD19-PerCP (clone HIB19), CD24-APC (clone ML5), CD38-FITC (clone HIT2), CD27-PECy7 (clone M-T271), IgG-PE-Cy7 (clone G18-145), IgD-BV421 (clone IA6-2), IgM-APC (clone MHM-88), and IgA-FITC (polyclonal, Southern Biotech). Zombie aqua (BioLegend, San Diego, CA) was used to differentiate live/dead lymphocytes.

Flow cytometry results were analyzed using FlowJo, version 10.7 (BD, Franklin Lakes, NJ). As illustrated in **Supplementary Figure 1**, switched (CD19^+^CD27^+^IgD^-^) and non-switched memory B cells (CD19^+^CD27^+^IgD^+^) were gated on the CD19^+^ subset and defined by their CD27 and IgD expression. Naïve B cells (CD19^+^CD24^+^CD38^-^), transitional B cells (CD19^+^CD24^hi^CD38^hi^), and plasmablasts (CD19^+^CD24^-^CD38^hi^) were gated on the CD19^+^ subset and defined by their CD24 and CD38 expression. IgG-producing B cells (CD19^+^IgG^+^), IgM-producing B cells (CD19^+^IgM^+^), and IgA-producing B cells (CD19^+^IgA^+^) were gated on the CD19^+^ subset and defined by their IgG, IgM, and IgA expression (data not shown). All B-cell subsets are shown as percent of total CD19^+^ cells.

### ELISA and multiplex assays for plasma biomarkers

Biomarkers associated with inflammation, macrophage, and lymphocyte activation were measured by magnetic bead-based multiplex according to the manufacturer’s instructions, including APRIL/TNFSF13, BAFF/TNFSF13B, sCD163, IL-2, IL-1β, IL-4, IL-5, IL-6, IL-10, IL-17A, IL-17F, IL-21, IL-22, IL-23, IL-25, IL-31, IL-33, IFN-γ, sCD40L, and TNF-α (Bio-Rad, Hercules, CA). Multiplex results were analyzed with Bio-Plex Manager Software (Bio-Rad, Hercules, CA). sCD14 was measured by ELISA according to the manufacturer’s instructions (R&D Systems, Minneapolis, MN) (18) and analyzed using Tecan’s Magellan™ Software (Tecan US, Morrisvillle, NC).

### Statistical analysis

The primary outcome of the study was tetanus, *HiB*, and *pertussis* IgG levels at 12 months of life. An unpaired non-parametric one-way ANOVA (Kruskal–Wallis), with Dunn’s *post hoc* test, was applied to detect changes in tetanus, *HiB*, and *pertussis* IgG levels, B-cell subsets, and plasma biomarker concentrations between time points. A Wilcoxon paired signed-rank test was used to detect changes in biomarker concentrations between mothers and infant CB samples. A Wilcoxon paired signed-rank test was used to examine transplacental transfer of biomarkers in the cross-sectional study involving mothers and their newborns. Spearman correlations were applied to define associations between plasma biomarkers. A Kruskal–Wallis analysis was performed to determine if sex, race, geographic location, GBS status, or feeding method affected vaccine IgG levels at 12 months. A linear mixed-effects model and a least absolute shrinkage and selection operator (LASSO) regression statistical model were applied to determine any associations between B-cell subsets (switched memory B cells, non-switched memory B cells, naïve B cells, transitional B cells, and plasmablasts), cytokine soluble factors (APRIL, BAFF, IL-2, sCD40L, IL-1β, IL-4, IL-17A, IL-21, IL-22, IL-25, IL-31, IL-33, IFN-γ, sCD163, and sCD14), and 12-month tetanus, *HiB*, and *pertussis* IgG levels. The LASSO regression model was fitted to input variables from each time point separately to identify soluble factors contributing to the vaccination outcome from samples collected at different times. A Benjamini–Hochberg correction with a false discovery rate (FDR) of 20% was used to correct for multiple comparisons in the simple linear regression and mixed-effects models. For biomarkers with values below the lower limit of detection (LLOD), a value one digit below the LLOD was assigned. Biomarkers were measured but not included in the analysis because more than 40% of the results below the lower limit of detection (LLOD) were IL-5, IL-6, IL-10, IL-23, and TNF-α. GraphPad Prism software version 9.4.1 (San Diego, CA) and the R language and environment for statistical computing was used for statistical analysis. Linear mixed-effects regression was performed using the lmerTest package, and LASSO regression was performed using the glmnet package in R.

## Results

### Study population

The longitudinal study included 82 term vaginally delivered healthy infants with a diverse racial background recruited from sites in Florida and North Carolina. Demographics of the study cohort are shown in **Table 1**. Infant sex was evenly distributed between female (52%) and male (48%) infants. Median gestational age of the cohort was 39 weeks (range 37–41 weeks). A third of the mothers (33%) tested positive for Group B Strep (GBS) prior to delivery, with all except one receiving antibiotics during delivery. Nearly half (49%) of infants were exclusively breast fed for the first 6 months of life, while 21% were exclusively formula fed, and 31% were fed a mixed diet of formula and breast milk. The cross-sectional study of 18 healthy mother/infant pairs included 67% Caucasian, 17% African American, and 39% Hispanic. The infants were 50% male and 50% female.

**Table 1:** Demographics of longitudinal healthy infant cohort.

Total *N* = 82	*N* (%)
Recruitment Location	
North Carolina	71 (86.6)
Florida	11 (13.4)
Mother’s Race and/or Ethnicity	
Caucasian	43 (52.4)
African American	25 (30.5)
Asian	7 (8.5)
Hispanic, Latin, or Spanish	3 (3.7)
Multiracial	2 (2.4)
Not provided	2 (2.4)
Infant Sex	
Female	43 (52.4)
Male	39 (47.6)
Infant’s Race and/or Ethnicity	
Caucasian	42 (51.2)
African American	25 (30.5)
Mixed race	8 (9.8)
Asian	5 (6.1)
Hispanic, Latin, or Spanish	2 (2.4)
Group B Streptococcus Screening Status	
Negative	54 (65.9)
Positive	27 (32.9)
Unknown	1 (1.2)
Feeding Method	
Breast fed	40 (48.8)
Mixed fed	25 (30.5)
Formula fed	17 (20.7)

All infants were born at term gestation (≥38 weeks) via vaginal delivery.

### *Bordetella pertussis* (*pertussis*)*, Haemophilus influenzae B*, and tetanus toxoid (tetanus) IgG levels over the first year of life

**Figure 2:**
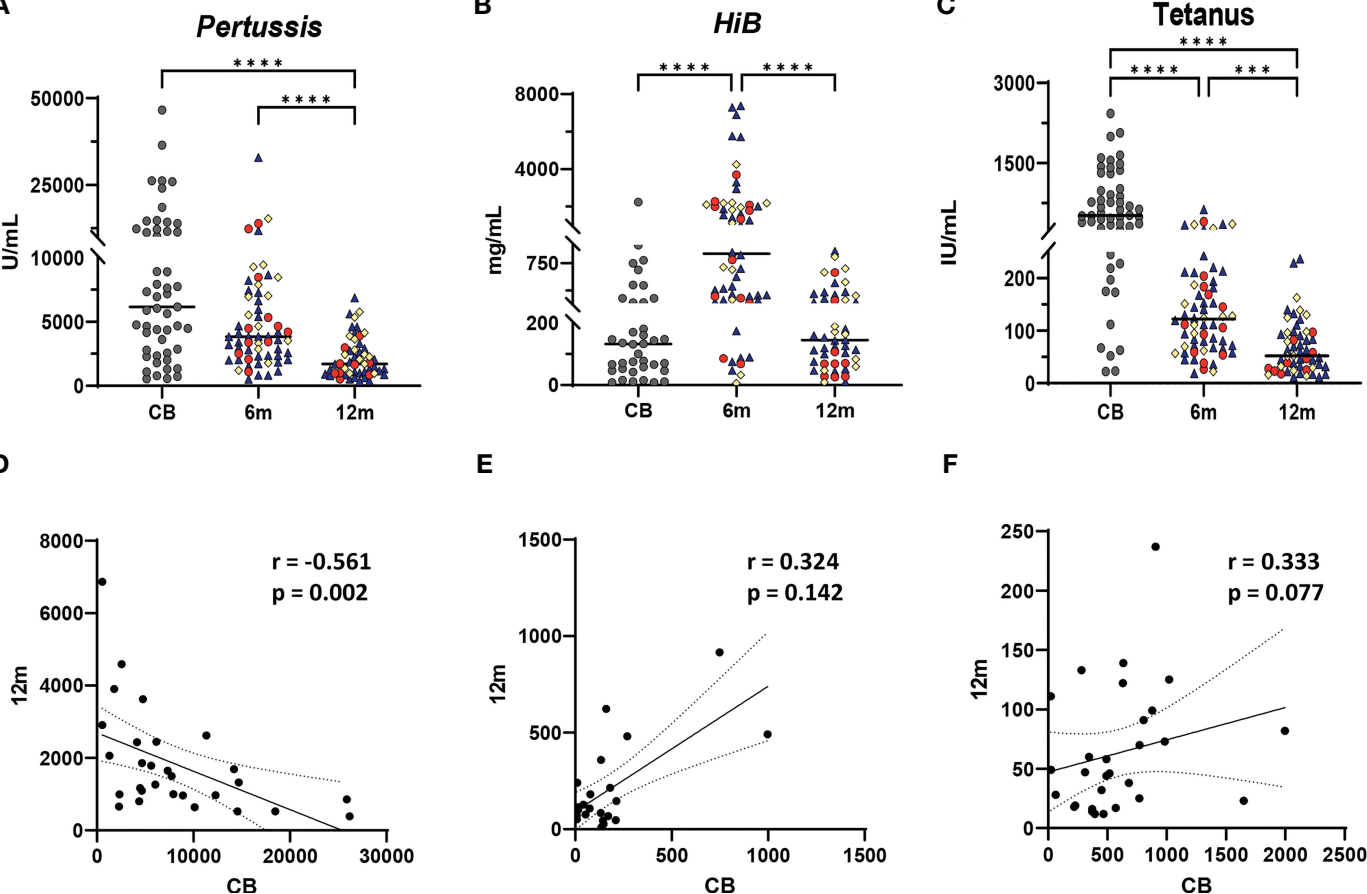
Vaccine IgG levels during the first year of life and relationship to vaccine-specific antibody levels in infants at 12 months. Vaccine IgG levels are shown from cord blood (CB), 6-month, and 12-month samples from each infant for *Bordetella pertussis* toxin (**A**, U/ml), *Haemophilus influenzae type B* (*HiB*) (**B**, mg/ml), and tetanus toxoid (**C**, IU/ml). A one-way ANOVA (Kruskal–Wallis) with Dunn’s *post hoc* test determined significant differences between CB, 6-month, and 12-month IgG levels. Significance is indicated by ***p ≤ 0.001, and ****p ≤ 0.0001. Gray dots represent CB infant samples. Shape colors represent feeding method during the first 6 months of life. Dark blue triangles designate breast-fed infants, red circles designate mixed-fed infants, and yellow diamonds designate formula-fed infants. Lines represent median values. Abbreviation CB is cord blood, m designates 6- and 12-month blood draws. **(D–F)** show Spearman correlations between cord blood (reflecting maternal IgG levels) and infant plasma IgG levels at 12 months for *Bordetella pertussis* toxin **(D)**, *HiB* **(E)**, and tetanus toxoid **(F)**. Each panel shows the *r* and *p*-values from the Spearman test. Solid line shows the best fit with dashed lines representing 95% confidence intervals.

All infants received *pertussis*, *HiB*, and tetanus immunizations at 2, 4, and 6 months of age. Antibody IgG levels were measured in CB prior to any immunizations, 7 to 14 days after the 6-month immunization, and just prior to the 12-month immunizations (**Figure 1**). CB IgG levels were highly variable across the cohort, reflecting the variable vaccination status of the mothers and resulting in passively acquired maternal antibodies in the newborns (**Figures 2A–C**) (19). Comparison of 6- and 12-month infant IgG levels showed a decline between immediate post-immunization IgG levels at 6 months and the IgG levels obtained at 12 months, just prior to the booster vaccinations.

Demographic variables including feeding method, maternal race, infant race, sex, and GBS status did not correlate with 6- or 12-month IgG levels of *pertussis*, *HiB*, or tetanus (data not shown). Higher *pertussis* CB IgG levels were weakly negatively correlated with *pertussis* IgG concentrations at 12 months (*r* = −0.5607, *p* = 0.002); in contrast, no significant correlations between CB and 12-month IgG levels to *HiB* and *tetanus* were detected (**Figures 2D–F**).

### Measurements of plasma biomarkers of immune development and cellular activation throughout the first year of life

**Figure 3:**
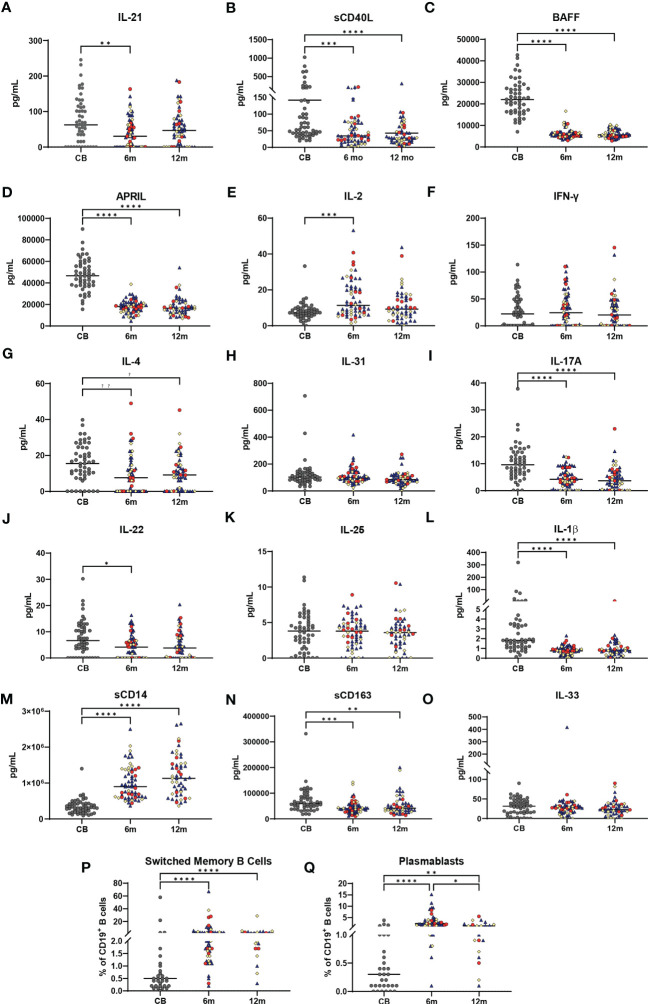
Changes in immune biomarkers and B-cell subsets across the first year of life. Plasma biomarker concentrations are shown in **(A–O)** with all values in pg/ml. Gray dots represent cord blood (CB) infant samples. Shape colors represent feeding method during the first 6 months of life. Dark blue triangles designate breast-fed infants, red circles designate mixed-fed infants, and yellow diamonds designate formula-fed infants. Lines represent median values. Abbreviation CB is cord blood, m designates 6- and 12-month blood draws. A one-way ANOVA (Kruskal–Wallis) with Dunn’s *post-hoc* test determined significant changes between CB, 6-month, and 12-month plasma soluble biomarkers or for B-cell subsets. **(P, Q)** show percentages of total CD19^+^ B cells for CD19^+^CD27^+^IgD^-^ switched memory B cells **(P)** and CD19^+^CD24^-^CD38^hi^ plasmablasts **(Q)**, in CB, 6-month, and 12-month blood samples from each infant. Significance is indicated by *p ≤ 0.05, **p ≤ 0.01, ***p ≤ 0.001, and ****p ≤ 0.0001. Lines represent median values.

Changes in plasma cytokines and soluble factors associated with innate, T-cell, and B-cell immunity were measured at each time point, while B-cell subsets were measured by flow cytometry. Biomarkers derived from the activated CD4 T cell and T follicular cell involved in immunoglobulin (Ig) class switch (IL-21, sCD40L) (10) and biomarkers involved in B-cell maturation and survival (BAFF and APRIL) (20) are shown in **Figures 3A–D**. These biomarker concentrations were highest in CB and declined during the first year of life. However, the concentrations, particularly in CB, were highly variable among infants. Biomarkers associated with Th1 T cells (IL-2 and IFN-γ, **Figures 3E, F**), Th2 (IL-4 and IL-31, **Figures 3G, H**), and Th17 T cells (IL-17A, IL-22, and IL-25, **Figures 3I–K**) (21–23) are also shown in **Figure 3**. IL-2 concentrations soon after vaccination (6 months) were higher than CB, while plasma IFN-γ concentrations did not change significantly before or after vaccination. IL-4 concentrations were highest in CB, but there was no difference in IL-31 concentrations in response to vaccination. IL-17A and IL-22 concentrations were highest in CB, declining at 6 and 12 months, while IL-25 concentrations did not significantly change with immunizations. Biomarkers associated with macrophage activation, including IL-1β, sCD14, and sCD163, and germinal center endothelial cell-derived IL-33 are shown in **Figures 3L–O** (24, 25). IL-1β, sCD14, and sCD163 displayed varying patterns in response to vaccine administration, whereas IL-33 displayed little change. A summary of the normal ranges for healthy infants for all the biomarkers is provided in **Supplementary Table 1**. CB contained lower percentages of B cells expressing IgG or IgA with decreases in naïve B cells but no change in transitional B-cell percentage across the first year of life (data not shown). The proportion of switched memory B cells and plasmablasts increased significantly comparing pre-vaccination CD19 B-cell percentages at birth (CB) to 7–14 days post vaccination (6 months), and this level was sustained throughout infancy (12 months) (**Figures 3P, Q**). Biomarker variations were not based on recruitment location, feeding method, race, sex, or GBS status (data not shown). CB contained lower percentages of B cells expressing IgG or IgA with decreases in naïve B cells but no change in transitional B-cell percentage across the first year of life (data not shown). In general, biomarker variations were independent of recruitment location, feeding method, race, sex, or GBS status (data not shown).

### Associations between IgG and plasma biomarkers levels at 6 months post vaccination

A linear mixed-effects model for the repeated measures revealed significant correlations between APRIL and IFN-γ with 12-month tetanus IgG levels and APRIL, BAFF, IL-21, IL-17A, and sCD14 with 12-month *pertussis* IgG levels (**Table 2**). A least absolute shrinkage and selection operator (LASSO) regression model, which uses L1 shrinkage to lessen the number of parameters in the model, was then used to identify potential biomarkers associated with outcomes at each sampling time point (**Table 2**) (26). LASSO regression modeling revealed multiple biomarkers and B-cell subsets that were associated with 12-month pertussis IgG levels. In CB samples, plasma IL-2, IL-17A, IL-31, sCD14, and switched memory B cells were positively associated, whereas APRIL, IL-33, non-switched memory B cells, and plasmablasts were negatively associated with 12-month *pertussis* IgG levels (**Table 2**). Only BAFF and IL-33 showed positive association at the early (6-month) post-vaccine time point, while none of the biomarkers measured at 12 months were associated with sustained *pertussis* IgG levels. Using the LASSO model, higher percentages of switched memory B cells in CB were associated with *HiB* IgG levels at 12 months. There was also a positive association with BAFF and a negative association with sCD14 at 12 months with *HiB* IgG concentrations (**Table 2**). Sustained *tetanus* IgG levels at 12 months were positively associated with CB concentrations of APRIL and sCD14 and negatively associated with IFN-γ. At the early post-immunizations time point, APRIL and IL-22 were positively associated with 12-month *tetanus* IgG levels, while plasmablasts were negatively associated. No 12-month biomarkers were significantly associated with *tetanus* IgG levels in the LASSO regression model.

**Table 2:** Plasma cytokine, soluble factors, and cell subsets correlating with 12-month post-vaccination *Pertussis*, *HiB*, or Tetanus IgG levels.

Statistical test	Cytokines/cell subsets with a significant *p*-value		
	*Pertussis*	*HiB*	Tetanus
Mixed effects (FDR 20%)	APRIL BAFF IL-21 IL-17A sCD14		APRIL IFN-γ
LASSO (pre-vaccine/CB)	IL-2**^pos^** IL-17A**^pos^** IL-31**^pos^** sCD14**^pos^** Switched memory B**^pos^** APRIL**^neg^** IL-33**^neg^** Non-switched memory B**^neg^** Plasmablasts**^neg^**	Switched memory B**^pos^**	sCD14**^pos^** APRIL**^pos^** IFN-γ**^neg^**
LASSO 6m (7–14 days post-vaccine)	BAFF**^pos^** IL-33**^pos^**		APRIL**^pos^** IL-22**^pos^** Plasmablasts**^neg^**
LASSO 12m (6 months post-vaccine)		BAFF**^pos^** sCD14**^neg^**	

FDR, false discovery rate; CB, cord blood; 6m, 6 months; 12m, 12 months.

LASSO statistical analysis: positive (pos) or negative (neg) correlations are shown.

### Comparisons of plasma biomarkers in mother/infant pairs

**Figure 4:**
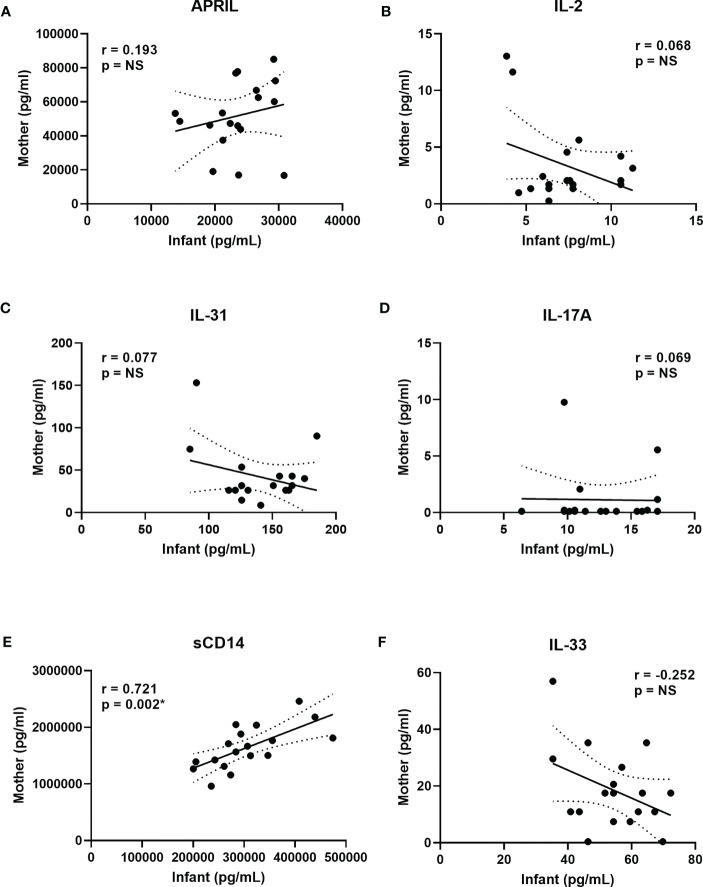
Correlations between mother and infant biomarkers to assess transplacental transfer. **(A–F)** show selected correlations between cord blood biomarker concentrations in the factors associated with vaccine IgG levels at 12 months (**Table 2**). Maternal plasma concentrations are on the *y* axis, and infant CB concentrations are on the *x* axis for APRIL **(A)**, IL-2 **(B)**, IL-31 **(C)**, IL-17A **(D)**, sCD14 **(E)**, and IL-33 **(F)**. Spearman correlations with respective *r* and *p*-values are shown in each panel. Solid lines show the best fit with dashed lines representing 95% confidence intervals. IFN-γ results are not shown since both mothers and infants had >40% of the results below the lower limit of detection (LLOD).

To determine if plasma CB biomarker concentrations associated with vaccine responses at 12 months were the result of transplacental transfer, plasma samples obtained less than 2 days prior to delivery from 18 healthy mothers were compared to the CB samples from their infants (**Figure 4**). The biomarkers shown in this figure included APRIL, IL-2, IL-31, IL-17A, sCD14, and IL-33. With the exception of sCD14 (**Figure 4E**), based on Spearman correlations, there were no significant associations between mother samples and CB samples. However, sCD14 demonstrated a significant positive correlation with higher concentrations in mothers reflected in their newborns, using a Wilcoxon paired signed-rank test, and plasma APRIL and sCD14 concentrations were higher in mothers than their infants; in contrast, IL-2, IFN-γ, IL-31, IL-17A, and IL-33 concentrations were higher in infants compared to their mothers (data not shown).

## Discussion

This study of healthy term infants fills an important gap in the understanding of the germinal center immune networks that shape vaccine response over the first year of life. Plasma bioprofiles assessed at key time points revealed the critical role of innate immunity biomarkers in sustained vaccine response at 12 months of age. Post-vaccination IgG levels are influenced by timing relative to immunizations and the nature of the vaccine antigens. The study design assessed bioprofiles at birth, within 2 weeks of the 6-month immunization battery, and 6 months later, prior to 12-month immunizations. These time points represent pre-immunization, early vaccine responses when germinal activity is greatest, and 6 months later as B-cell memory is established (27–29). While diverse in sex, ethnicity, and feeding patterns, the study cohort consisted of healthy term vaginally delivered newborns. The demographic composition of the study cohort, feeding methods, or maternal group B Strep status at birth did not appear to affect immunization IgG levels, although the statistical power of this small study is limited. There was a weak negative correlation between *pertussis* IgG levels in CB and *pertussis* IgG levels in the infants at 12 months, reflecting previously reported interference by passively acquired maternal IgG on vaccine response (30, 31). The effect of passively acquired maternal IgG did not affect *HiB* or tetanus IgG levels (12, 30, 31). Furthermore, CB vaccine IgG levels were highly variable across the cohort, perhaps reflecting differences in vaccine status in the mothers. Switched memory B cells develop quickly over the first 6 months of life and are maintained, with plasmablasts at their peak immediately after the battery of 6-month immunizations (29). The results indicate that the study cohort displayed the expected post-immunization patterns of B-cell development to allow for an assessment of the relationship between immune biomarkers and immunization responses in healthy infants (3, 4, 8, 32).

Multiple biomarkers (IL-21, sCD40L, BAFF, APRIL, IL-4, IL-17A, IL-22, IL-1β, and sCD163) were elevated at birth compared to 6 months of age. The factors included were typically associated with Ig class switch and germinal center development, such as BAFF, APRIL, IL-21, and sCD40L (33). Correlations of the concentration of paired mother and infant samples at birth indicated that these factors were unlikely simply the result of transplacental transfer but rather reflect the maternal/fetal interface. Multiple studies of maternal and CB cytokine levels show that perturbations in plasma factors including BAFF, TNF-α, and sCD40L may play a role in the pathogenesis of conditions such as pre-eclampsia (34–37). Furthermore, elevated concentrations of BAFF in newborns may be due to secretion by maternal-derived decidual stromal cells stimulated by IFN-γ and IFN-α (38–40). While higher BAFF concentrations at birth are negatively associated with the development of allergic disease (41), the association of BAFF, APRIL, and IL-21 concentrations with vaccine responses is a novel finding from our study. In contrast to BAFF or APRIL, elevated sCD14 in mothers was positively associated with elevated sCD14 in their newborns. This result may indicate transplacental transfer of sCD14 or, alternatively, the effect of inflammatory factors such as LPS from the mother activating TLR4 and release of sCD14 in the newborn (19, 42). Taken together, these results indicate a surprisingly high concentration of biomarkers associated with germinal center and macrophage activity at the time of birth. Increasing sCD14 concentrations from birth to 6 and 12 months may reflect microbial translocation following the establishment of the microbiome (43, 44). Elevated CB IL-4 and IL-22 likely reflects inherent bias towards intrauterine Th2 polarization and IL-22 production (22). The high concentrations of CB IL-17A supports the early emergence of Th17 cell polarization during infancy (45–47). Compared to CB, plasma IL-2 concentrations were elevated after the 6-month immunizations, but other biomarkers known to play key roles in immune development, such as in IFN-γ, IL-31, IL-25, and IL-33, displayed little variability across time points. While it is difficult to conclude whether changes in plasma biomarkers from birth to 6 months result from immunizations or natural immune development, the dynamic changes in immune-based biomarkers in early life have implications for sustaining post-vaccine antibody responses later in infancy.

**Figure 5:**
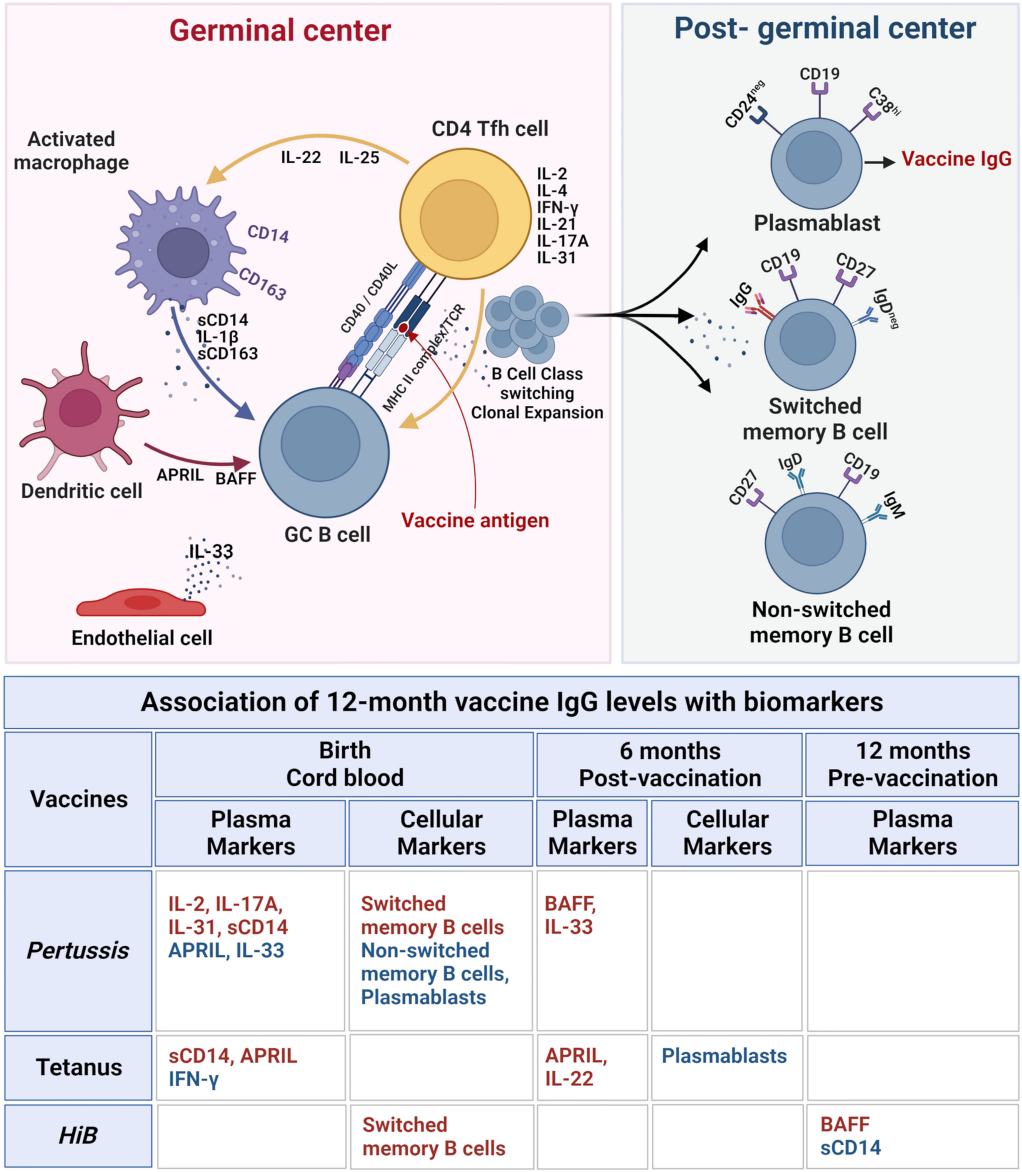
Immune biomarkers associated with sustained post-immunization IgG antibody levels at 12 months in healthy infants. Top figure: The biomarkers and cellular interactions between T follicular helper cells (Tfh), B cells, macrophages, dendritic cells, and endothelial cells within the lymphoid germinal centers are shown to represent the cytokines and soluble factors that influence vaccine responses as measured by vaccine IgG levels. Following vaccine antigen activation, B cells undergo class switch and clonal expansion and develop into post-germinal center plasmablasts and switched and non-switched memory B cells. The lower table lists the soluble and cellular biomarkers associated with vaccine-specific 12 months IgG levels with biomarkers at each blood sample time point associated with vaccine response (birth cord blood, 6 months post-vaccination, and 12 months pre-vaccination). Positive associations with vaccine IgG levels are highlighted in red, and negative associations are in blue. All correlations are based on LASSO analysis results shown in **Table 2**. Upper diagram created with BioRender.com.

The three vaccines studied were T-cell dependent or conjugated protein antigens with variable efficacy. After three administrations, *tetanus* toxoid elicits nearly 100% protection, while conjugated *HiB* vaccine is 94% effective; in contrast, *pertussis* is only 80%–90% effective after five administrations (48, 49). Analyzed together, these vaccines allow comparisons between immunogens and biomarkers in sustaining specific vaccine antibody responses (8). LASSO was used to identify sparse features associated with outcome IgG levels for each sample time point. Using this model, CB APRIL and sCD14 concentrations were associated with higher tetanus IgG levels at 12 months. TLR4 adjuvants such as LT-K63r enhance B-cell survival and may enhance long-lived humoral immunity to vaccines like *pertussis* and *tetanus* (11, 50, 51). In contrast, IFN-γ had a negative association with *tetanus* IgG levels. LASSO modeling also revealed positive association between *pertussis* IgG levels with CB concentrations of T-cell-derived cytokines IL-2, IL-17A, and IL-31, but in contrast to tetanus, APRIL was negatively associated with *pertussis*, as was IL-33. In addition, LASSO modeling pointed to a positive association between higher concentrations of switched memory B cells at birth with *pertussis* and *HiB* IgG levels, suggesting that enhanced early development of B-cell Ig class switch may sustain vaccine responses at 12 months (52–57). Taken together, biomarkers known to reflect early events in B-cell development shape sustained IgG levels for at least 6 months following immunization. The study was designed to measure maximal B-cell activation by measuring cellular and plasma biomarkers 7 to 14 days after the 6-month immunization battery. At this time point, BAFF and IL-33 were associated with higher 12-month pertussis IgG levels, and APRIL and IL-22 were associated with tetanus. Paradoxically, plasmablast B cells, reflecting B-cell activation, were negatively associated with pertussis vaccine response at birth and tetanus IgG levels at 6 months, indicating that immune regulatory mechanisms activated during early life may dampen sustained vaccine responses (58, 59). Even though biomarker- and vaccine-specific responses showed extensive variation, factors involved in germinal center development and Ig class switch were most consistently associated with sustained antibody responses 6 months after vaccination (12 months of age) (20, 60, 61). A conceptual framework to summarize the relationship between the steps involved in memory B-cell development and sustained antibody production and the contributions of the cellular and soluble factors within the germinal centers is illustrated in **Figure 5**. Biomarker concentrations early after multiple vaccine administrations (6 months) were not strong predictors of antibody concentrations 6 months later. However, CB biomarkers appear to have a more dynamic association with sustained antibody concentrations for *pertussis* and *tetanus*. Although the study has a small cohort and focused primarily on plasma-based biomarkers, it is significant as it is one of the few longitudinal studies that discussed the complex relationship between biomarkers of human immune development relative to post-vaccine antibody levels. Many of the biomarkers that shape early immune responses are concentrated in lymphoid tissues and are below the limit of detection for plasma-based assays. Consequently, conclusions regarding the precise role of cytokines, chemokines, and soluble factors can only be inferred. Furthermore, variability in assays measuring antibody concentrations, blood B-cell sub-populations, and plasma biomarkers require the application of multiple statistical methods to discover associations within sustained vaccine antibody levels. The results reveal that even with differences among specific immunogens, sustained B-cell immunity is significantly influenced by early life immune dynamics detectable at birth. Our study has broad applicability to many maternal/fetal conditions and fills gaps in our understanding of vaccine responses in early life (62, 63). Conditions impacting neonatal immunity such as pre-eclampsia, chorioamnionitis, and maternal HIV infection are likely to affect early immune responses to vaccines (63, 64). Defining the immune bioprofile of healthy infants with respect to immunization response provides the foundation to examine the effects of maternal inflammatory disorders on at-risk infants who might benefit from altered vaccine strategies to optimize effectiveness.

## Data availability statement

The original contributions presented in the study are included in the article/**Supplementary Material**. Further inquiries can be directed to the corresponding author.

## Ethics statement

The studies involving human participants were reviewed and approved by Duke University Health Systems Institutional Review Board. Written informed consent to participate in this study was provided by the participants’ legal guardian/next of kin.

## Author contributions

Study concept and design: GA, MG, JS. Data acquisition and analysis: CB, GV, BF, JK-C, KD, LS. All authors contributed to the article and approved the submitted version.

## Supplementary material

Supplementary Figure 1 Flow cytometry gating of B-cell subsets. **(A)** To measure the percentages of the switched (CD19^+^CD27^+^IgD^-^) and non-switched memory B cells (CD19^+^CD27^+^IgD^+^) subsets, lymphocytes, defined by their forward and side scatter properties, were first gated on CD19^+^ and then defined by CD27 and IgD expression. To measure the percentages of naïve B cells (CD19^+^CD24^+^CD38^-^), transitional B cells (CD19^+^CD24^hi^CD38^hi^), and plasmablasts (CD19^+^CD24^-^CD38^hi^) subsets, lymphocytes were first gated on the CD19^+^ and then by CD24 and CD38 expression. **(B)** To measure the percentages of IgG-expressing B cells (CD19^+^IgG^+^), IgM B cells (CD19^+^IgM^+^), and IgA B cells (CD19^+^IgA^+^), lymphocytes were gated on CD19^+^ subset and defined by their IgG, IgM, and IgA expression. All of the B cell results are shown as percent of total CD19^+^ cells.
