# Supplementary figures and images for: Biomarkers detected in cord blood predict vaccine responses in young infants
Source: Front Immunol. 2023 May 12;14:1152538. doi: 10.3389/fimmu.2023.1152538 (PMC10213698; doi:10.3389/fimmu.2023.1152538)

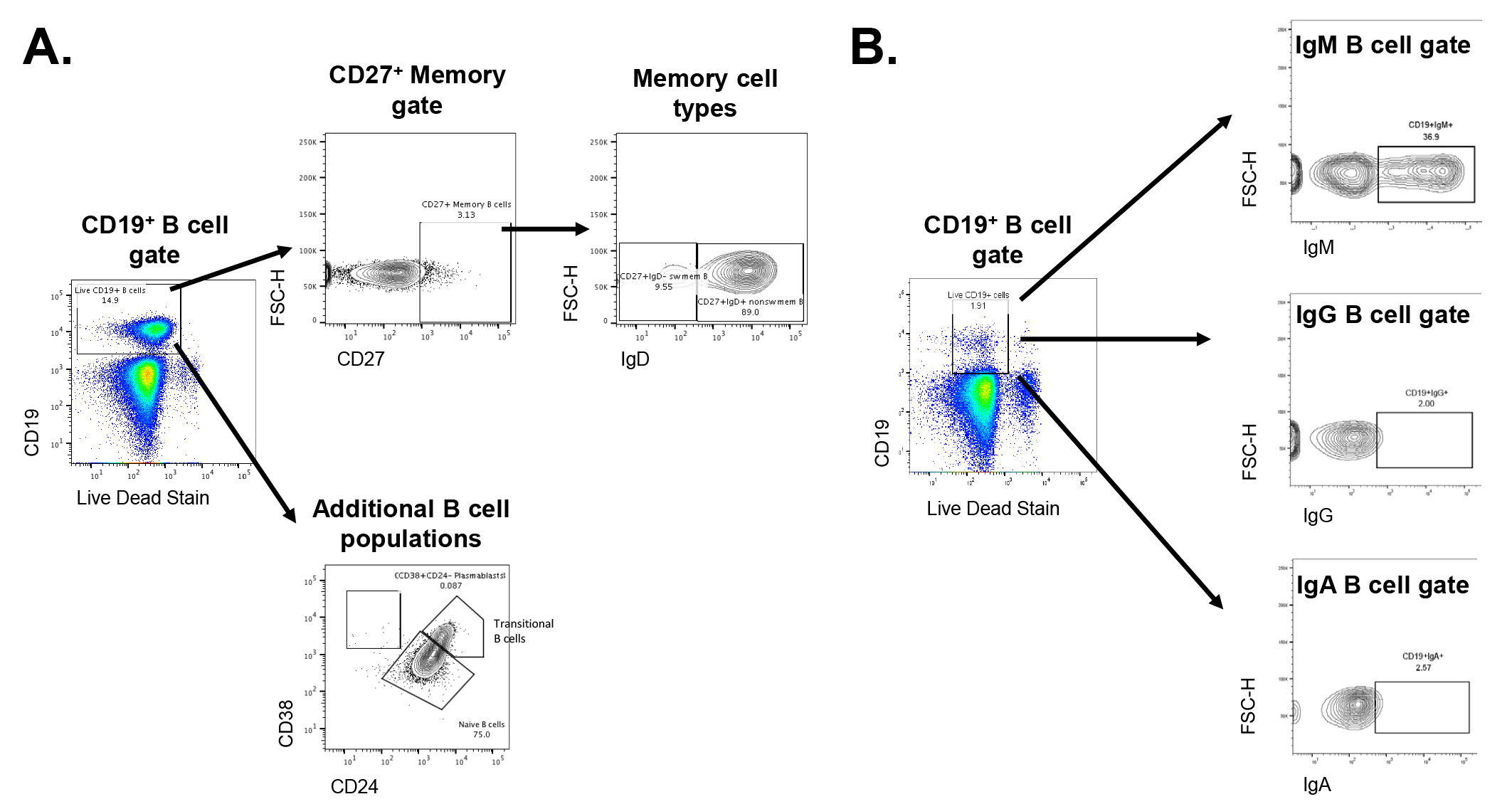

Supplement: Supplementary Figure 1 — Flow cytometry gating of B-cell subsets. (A) To measure the percentages of the switched (CD19+CD27+IgD-) and non-switched memory B cells (CD19+CD27+IgD+) subsets, lymphocytes, defined by their forward and side scatter properties, were first gated on CD19+ and then defined by CD27 and IgD expression. To measure the percentages of naïve B cells (CD19+CD24+CD38-), transitional B cells (CD19+CD24hiCD38hi), and plasmablasts (CD19+CD24-CD38hi) subsets, lymphocytes were first gated on the CD19+ and then by CD24 and CD38 expression. (B) To measure the percentages of IgG-expressing B cells (CD19+IgG+), IgM B cells (CD19+IgM+), and IgA B cells (CD19+IgA+), lymphocytes were gated on CD19+ subset and defined by their IgG, IgM, and IgA expression. All of the B cell results are shown as percent of total CD19+ cells. [file Image_1.tif]
