# Supplementary material for: Biomarkers detected in cord blood predict vaccine responses in young infants
Source: Front Immunol. 2023 May 12;14:1152538. doi: 10.3389/fimmu.2023.1152538 (PMC10213698; doi:10.3389/fimmu.2023.1152538)
Supplement: Supplementary file 2 [file Table_1.docx]

**Supplemental Table 1. Plasma soluble factors and cytokine levels (pg/ml) in infants over the first year of life.**

|  | **Infants** | | | | | |
| --- | --- | --- | --- | --- | --- | --- |
|  | **Cord Blood** | | **6 months** | | **12 months** | |
| **Biomarker** | **Median** | **IQR^a^** | **Median** | **IQR^a^** | **Median** | **IQR^a^** |
| **IL-21** | 62.7 | 15.5-114.5 | 30.4* | 0.08-63.5 | 46.7 | 4.40-78.03 |
| **sCD40L** | 56.6 | 41.4-139.6 | 34.5* | 19.04-74.05 | 29.1* | 19.5-55.4 |
| **BAFF** | 22,015 | 16,424-  27,218 | 5,655* | 4,912-6,772 | 5,631* | 4,800-  7,659 |
| **APRIL** | 46,703 | 37,822-  56,973 | 18,126* | 13,806-21,974 | 16,447* | 12,504-  22,101 |
| **IL-2** | 7.4 | 5.6-9.7 | 11.4* | 7.2-21.7 | 9.3 | 5.4-15.8 |
| **IFN-ɣ** | 22.2 | 0.4-45.4 | 24.4 | 0.4-54.3 | 20.2 | 0.4-42.8 |
| **IL-4** | 15.5 | 9.04-24.4 | 7.6* | 0.1-14.7 | 9.2* | 0.1-14.7 |
| **IL-31** | 103.2 | 77.5-134.7 | 95.9 | 73.5-134.4 | 84.3 | 66-114.3 |
| **IL-17A** | 9.6 | 6.2-14 | 4.2* | 1.5-7.8 | 3.7* | 1.0-6.8 |
| **IL-22** | 6.7 | 2.5-11.7 | 4.2* | 0.03-6.6 | 3.8 | 0.03-8.4 |
| **IL-25** | 3.8 | 1.8-5.4 | 3.8 | 2.2-5 | 3.6 | 2.0-4.5 |
| **IL-1β** | 1.8 | 1.2-3.5 | 0.8* | 0.4-1.1 | 0.8* | 0.4-1.1 |
| **sCD14** | 310,220 | 203,495-  427,995 | 901,005* | 607,018-1,358,926 | 1,130,300* | 719,860-  1,535,815 |
| **sCD163** | 59,479 | 44,953-  85,525 | 39,755* | 28,268-52,959 | 41,624* | 29,928-  61,484 |
| **IL-33** | 31.5 | 15.3-44.3 | 26.8 | 14.9-33.8 | 22.6 | 13.1-34.6 |

*: Indicates 6-month or 12-month levels are significantly different from CB level (One-way ANOVA (Kruskal-Wallis) with Dunn’s post hoc test; p < 0.05).

**^a^**: IQR (Interquartile Range)
